# Patterns of emotional expression during the formation of egocentric awareness in early childhood: a case study

**DOI:** 10.3389/fpsyg.2026.1756750

**Published:** 2026-04-20

**Authors:** Lin Guo, Zhenyao Quan, Chengyu Nan

**Affiliations:** 1 School of Finance, Southwestern University of Finance and Economics, Chengdu, China; 2 College of Foreign Languages, Yanbian University, Yanji, China

**Keywords:** affective dialogue, affective monologue, egocentric awareness, emotional expression, other-oriented feedback

## Abstract

Emotional expression is a fundamental dimension of children’s social development and is closely linked to psychological processes, language competence, and self-awareness. Drawing on developmental psychology and cognitive linguistics, this study traces how a two-year-old Mandarin-speaking child develops self-recognition and egocentric awareness. Through naturalistic observation and discourse analysis, this study delineates the developmental stages of emotional expression, examines the dynamic interplay between self-awareness and language awareness across stages, and further explores how affective dialogue fosters the differentiated development of egocentric awareness. The findings indicate that egocentric awareness unfolds along a developmental continuum from affective monologue to affective dialogue. In the monologue stage, self-recognition and language awareness emerge concurrently; in the dialogue stage, egocentric awareness co-develops with language-mediated embodied thinking and an increasing demand for interpersonal feedback. Bidirectional affective expression at this stage appears to promote the development of children’s general cognitive abilities. Grounded in authentic linguistic and behavioral data, this study offers a preliminary exploration of emotional expression patterns during children’s mental maturation and provides tentative insights for constructing an “emotion-language” co-development model.

## 1 Introduction

How emotional expression evolves with the maturation of self-representation remains a “black-box” question shared by developmental psychology and cognitive linguistics (Bates et al., 1979; Tomasello, 2003). Traditional research has primarily focused on two ends of emotion: the recognition and expression of basic emotions, and the emergence of higher-order self-conscious emotions. However, systematic, micro-level accounts of how emotional expression develops between these two poles remain limited. By observing how a two-year-old Mandarin-speaking child adapts nursery rhymes to express emotions and elicit social feedback, this study investigates the stage-specific changes and characteristics of emotional expression in early childhood.

### 1.1 Theoretical foundations of emotional expression and early cognitive development

Research on children’s emotional development generally holds that primary emotional expressions, emerging as early as infancy, initially serve to satisfy internal needs and support emotional discharge (Denham, 1998). During this period, children’s emotional expression and cognitive development are closely intertwined and co-develop. Jean Piaget’s theory of cognitive development posits that early childhood is dominated by egocentric thinking (Piaget, 1952), whereby children have difficulty understanding the world from others’ perspectives. The development of self-awareness is a critical milestone in this process. Beulah Amsterdam’s (1972) “mirror self-recognition test” experiment demonstrates that infants typically acquire self-recognition between 18 and 24 months, which enables them to perceive themselves as distinct from the external environment. The acquisition of this ability marks the emergence of self-awareness. Thereafter, children’s emotional expression becomes increasingly complex and progresses alongside cognitive development. Piaget (1952) further emphasized that, under egocentric thinking, children’s emotional experiences and expressions remain largely self-centered, thereby laying a foundation for the subsequent development of social emotions.

### 1.2 Language as a medium for emotional expression and cognition

From the perspective of cognitive linguistics, language is not merely a tool for communication but a medium for cognitive structuring and emotional regulation (Lakoff and Johnson, 1980). In children’s development, language acquisition significantly expands the scope and complexity of emotional expression. Children’s intentional use and adaptation of language go beyond simple imitation; it demonstrates their active reorganization of linguistic forms in accordance with their emotional experiences (Li, 1995). Language thus becomes a central instrument through which children explore the world, manage emotions, and participate in social interaction.

Overall, prior research typically distinguishes between primary and higher-order stages of emotional development, yet the transitions and intermediate processes between these stages remain insufficiently examined. Moreover, large-sample developmental studies tend to overlook the momentary, individualized events that reveal the dynamic coupling between emotional and language development. Therefore, by examining a two-year-old child’s creative adaptation of nursery rhymes and creative use of language to express emotion, this study offers a micro-level account of the development of egocentric awareness and its stage-specific features. Such an approach is well suited to capturing fine-grained developmental processes that are difficult to detect in large-sample designs.

## 2 Materials and methods

### 2.1 Participants

Dundun (pseudonym), a two-year-old girl, is the focal child in this study. Preliminary informal observations suggested that her patterns of emotional expression in both verbal and behavioral modalities were sufficiently representative to support an in-depth case analysis. In addition, her parents and maternal grandmother were interviewed to provide first-hand accounts and retrospective descriptions of research-related events.

### 2.2 Research questions

1. What stage-specific features of emotional expression emerge during the development of self-recognition and egocentric awareness?
2. How do self-awareness and language awareness interact at each stage?
3. Why does bidirectional affective interaction stimulate the differentiated development of egocentric awareness?

### 2.3 Methodology

#### 2.3.1 Naturalistic observation

The child was observed in her natural home environment, with particular attention to spontaneously occurring episodes of emotional expression and language use. Observations focused on (i) spontaneous verbal expressions and improvised singing, (ii) nonverbal behaviors accompanying emotional expression, (iii) antecedents and consequences of expressive episodes, (iv) complete sequences of interactions with family members.

To minimize potential observer effects and reactivity, observations were conducted by familiar caregivers who were regularly present in the child’s daily environment. The child was accustomed to their presence, and no intrusive recording procedures were introduced that might disrupt routine interactions. In addition, observation was integrated into everyday activities, allowing behaviors to be captured in a naturalistic context.

#### 2.3.2 Interviews

The father was interviewed about the child’s creative language use and emotional expression in daily contexts, and the mother about broader aspects of the child’s language development and manifestations of self-awareness.

#### 2.3.3 Retrospective method

The maternal grandmother was invited to recall and offer detailed retrospective accounts of her daily interactions with the child, as well as the child’s adaptation of the “One Cent” song, including contextual, motivational and interactional information. Cases 2, 3 and 4 reported in this study were derived from these retrospective accounts.

### 2.4 Linguistic and behavioral data

The key observational cases are summarized in Table 1. The full transcripts of the cases are provided in the Supplementary material.

**Table 1:** Cases illustrating the developmental progression of emotional expression from affective monologue to affective dialogue (18–28 months).

Case	Age (months)	Context	Observed behavior (original utterance)
1	18	Nursery rhyme adaptation	Hummed “A Mama’s love is the best in the world.”
	19		Replaced “mama” with “papa” in the opening line.
	22		Full substitution (replaced all three “mamas” with “papa,” with father absent).
	23		Replaced with “gege” (absent).
2	26	Daily conversation (reference understanding)	Identified “mama,” “papa,” and “treasure” as self-related, but failed to interpret “happy” and “embrace.”
3	27	Daily interaction	“Mama is here” (pats chest); when asked “What is mama’s embrace?,” responds “Mama’s embrace is mama.”
4	28	Nursery rhyme adaptation (situational)	Replaced “policeman” with “grandmother” in “One Cent,” directing the performance toward her and anticipating her response.
5	28	Daily interaction (emotionally salient event)	In response to the melting ice cream, expressed frustration by saying: “Grandpa Sun is too bossy! Let the moon come out!”

### 2.5 Procedures

The study spanned ten months and consisted of three phases.

#### 2.5.1 Phase 1 (month 1): preparation

During the first month, informed consent was obtained. Initial naturalistic observations were conducted over multiple sessions in the home environment, and a semi-structured interview with the parents was carried out to collect the child’s developmental history and early emotional expression events. In addition, family members were given general guidelines outlining the key behaviors to be observed, with a focus on the child’s spontaneous emotional expressions, language use, and associated behavioral contexts.

#### 2.5.2 Phase 2 (months 2–9): longitudinal naturalistic observation

During the subsequent 8 months, Dundun’s caregivers engaged in ongoing naturalistic observation of her spontaneously occurring behaviors in daily life. Observations focused on song adaptations, creative metaphors, and related expressive behaviors. In parallel, monthly interviews were conducted with family members to collect representative cases of the child’s linguistic and behavioral performance and to provide contextual information for subsequent analysis.

#### 2.5.3 Phase 3 (month 10): data integration and analysis

In the final month, all observation notes, interview data, and retrospective accounts were transcribed, and multiple data sources were systematically cross-validated.

## 3 Results

### 3.1 Children’s transition from self-recognition to egocentric awareness involves a progression from affective monologue to affective dialogue

As children develop from self-recognition toward egocentric awareness, their emotional expression shifts from unidirectional output to interaction-oriented dialogue. In the stage of self-centered unidirectional output, emotional expression flows outward from the self and primarily serves to release affect or communicate internal needs, with little attention to others’ feedback (as in Case 1). This form of unidirectional self-recognition and expression is conceptualized in this study as *Affective Monologue*. As development progresses, children’s emotional expression evolves into a socially oriented form that actively seeks interaction and anticipates responses. Emotional expression becomes bidirectional, extending between self and others, while remaining self-centered rather than involving full perspective-taking (as in Case 2). This process of self-expression and social feedback is conceptualized in this study as *Affective Dialogue*.

### 3.2 Children’s language awareness progresses from referential to conceptual symbols across the stages of affective monologue and affective dialogue

During the stages of affective monologue and affective dialogue, children are in a transitional phase of cognitive development from self-recognition to egocentric awareness. Over a ten-month longitudinal observation, Dundun’s verbal productions illustrate this progression. At earlier stages, her language use was primarily referential and grounded in concrete entities, as reflected in utterances such as replacing “papa” in song lyrics, identifying “papa” as her father, and stating “I am mama’s baby.” At the same time, although she could fluently reproduce songs, she showed limited understanding of abstract terms such as “happiness,” “world,” and “embrace.” By 27 months of age, Dundun pointed to her chest and said, “Mama is here.” These observations highlight differences in children’s language production and acquisition across the stages of affective monologue and affective dialogue, and provide a basis for analyzing developmental characteristics in the acquisition of linguistic symbols, the construction of language awareness, and their cognitive underpinnings during the transition from self-recognition to early egocentric awareness.

### 3.3 The transition from affective monologue to affective dialogue promotes the differentiated development of egocentric awareness

In the stage of affective monologue, children’s language and emotional expression primarily take the form of outward projections of internal states. In contrast, during the stage of affective dialogue, children’s expressive needs shift: they not only seek to express their own emotions but also expect responses and feedback from others. In other words, their attentional focus moves from unidirectional expression to a bidirectional pattern of expression and feedback. This shift enables children to recognize that their expressions can elicit attention and responses from their interlocutors. Thus, the transition from monologue to dialogue involves not merely a change in the object of emotional expression, but a restructuring of consciousness itself.

Utterances such as those in Case 4 and Case 5 illustrate how bidirectional emotional interaction can generate multi-dimensional projections of egocentric awareness. Specifically, in Case 4, the child adapts nursery rhyme lyrics by replacing “policeman” with “grandmother,” thereby identifying an emotional object from whom responses and feedback can be obtained. In doing so, she recognizes that “policeman” and “grandmother,” despite their differing social roles, can be grouped within the same relational category. In Case 5, when the child was told that the ice cream had melted, she did not continue to request it; instead, her attention shifted from the object (“ice cream”) to its underlying cause. This “why”-oriented shift appears to initiate a causal reasoning sequence—from “melting” to “heat,” and ultimately to the sun as its source—resulting in the creative utterance “The sun is too bossy!” This process reflects a transition from immediate desire to causal inquiry, during which the child’s thinking extends from a concrete object to a broader functional and environmental context. Across these cases, Dundun’s thinking progresses from “one cent” to “policeman” and then to “grandmother,” and from a desire for “ice cream” to the sun and its function, forming multi-dimensional associative links and supporting the differentiated development of egocentric awareness.

## 4 Discussion

This study finds that during early psychological development, particularly in the stages of self-recognition and the emergence of self-awareness, children display an increasing tendency to express emotions, and their expressive strategies exhibit stage-specific transitions. Specifically, children appear to progress from affective monologue to affective dialogue. Although these findings have not been systematically substantiated, the core observations are consistent with established research in developmental psychology, particularly with respect to egocentrism, emotional socialization, and the internalization of egocentric speech. Based on observations of Dundun’s linguistic and emotional behaviors, the following discussion is presented.

### 4.1 Affective monologue and self-recognition (before 26 months)

In Case 1, Dundun replaced “mama” with “papa” or “gege” (older brother) in the nursery rhyme “Only Mama is Good in this World,” expressing her longing for her father and her reluctance to separate from her brother. Notably, when she sang these modified lyrics, neither “papa” nor “gege” was present. This case shows that Dundun’s singing at this stage served as a means of expressing her own internal emotions, with emotional release as the central purpose. Her psychological development can be characterized as remaining at the self-recognition stage, corresponding to affective monologue and consistent with Amsterdam’s mirror self-recognition paradigm. Just as infants recognize that the image in the mirror represents the self, Dundun’s modification of song lyrics can be interpreted as a form of affective self-referencing. She expresses her emotions through singing without requiring an audience; her attention is fully centered on the correspondence between “the self” and her own emotions. In this sense, her singing functions as an “affective mirror” of her inner state, through which she engages in a form of self-directed affective expression, reinforcing feelings such as missing her father or reluctance to part from her brother.

### 4.2 Affective dialogue and the establishment of egocentric awareness (27 months)

In Case 4, Dundun replaced the original lyric “give it to the policeman” in “One Cent” with “give it to grandma,” walked over to her grandmother, and sang to her while maintaining steady eye contact, which indicates her anticipation of praise. This case indicates that Dundun’s emotional expression had shifted from simple self-directed release to an expectation of response from her grandmother, reflecting a clear interactional intent. Her behavior forms a bidirectional interactional sequence of “expression → feedback → emotional satisfaction,” characterized by feedback anticipation and an affective feedback loop. Her sustained eye gaze (looking directly at her grandmother) and subsequent action (throwing herself into her grandmother’s arms) further suggest that emotional expression was experienced as a reciprocal cycle of communication and feedback. This pattern marks the emergence of bidirectional emotional interaction and the initial formation of egocentric awareness. Such behaviors are consistent with Lev Vygotsky’s (1987) description of “social speech” development. This shift from singing for herself to singing for her grandmother and awaiting her response reflects a developmental transition from “the self” in the stage of affective monologue to the establishment of a “self-other” relation in the stage of affective dialogue. By modifying the lyrics to “hand it to grandmother,” Dundun’s expression acquires an interpersonal orientation. The target of her expression is no longer an absent figure serving as an object of emotional projection (e.g., her father or older brother), but a present interlocutor capable of immediate social engagement (her grandmother). This shift is consistent with Vygotsky’s (1978) central proposition that higher psychological functions, including self-awareness, emerge through social interaction. Through repeated cycles of “expression → feedback → emotional satisfaction,” Dundun gradually internalizes external social relations (the intimate bond between herself and her grandmother) into internal psychological structures, contributing to a sense of being valued and loved.

Nevertheless, alternative interpretations of this behavior should be considered. For instance, Dundun’s actions may partly reflect imitation of familiar interactional patterns or a tendency to seek immediate reward. These interpretations are plausible, as imitation and reinforcement play well-established roles in early development. However, rather than contradicting the present interpretation, these processes may be understood as components of the broader mechanism of affective dialogue. In other words, imitation and reward-seeking behaviors are themselves oriented toward obtaining social feedback. What distinguishes the behaviors observed in this study is not the presence of these processes per se, but the way in which the child flexibly integrates them with symbolic modification, relational re-mapping, and context-sensitive expression. Therefore, while imitation and reward likely contribute to the observed behaviors, the evidence suggests that they operate within a more complex interactional framework, in which affective dialogue supports the development of egocentric awareness and early cognitive structuring.

### 4.3 Language awareness and characteristics of language acquisition across the stages of affective monologue and affective dialogue

Around the age of two, children enter an important developmental period for cognitive and language acquisition, which overlaps with the emergence of mirror self-recognition and reflects the coordinated influence of biological maturation and social cognition (Lenneberg, 1967). The emergence of self-recognition is associated with increasing demands for emotional expression, leading to a rapid expansion in the range of emotional states. As a result, the facial expressions and gestures typical of infancy become insufficient for expressing this expanding emotional repertoire, prompting children to attend to and imitate adult language (Lindquist et al., 2015). During this period, complex self-related emotional representations and language production not only develop in parallel but also interact dynamically.

#### 4.3.1 Language awareness and acquisition during the stage of affective monologue

A daily conversation with the maternal grandmother at 26 months of age (Case 2) showed that Dundun clearly understood that “papa” in the nursery rhyme referred to her own father. This indicates that she had not only acquired basic referential terms but had also begun to establish a cognitive link between linguistic symbols and their real-world referents. Utterances such as “my papa” and “I am mama’s baby” further suggest that she had developed the foundational language awareness and cognitive bases for symbol acquisition, namely a “self-other-object” triadic relation (Morales et al., 2000).

However, when linguistic forms referred to abstract concepts instead of concrete, perceptible entities, such as “world,” “embrace”, or “happiness,” she was still unable to grasp their meanings. This suggests that, during the stage of affective monologue, children’s cognition remains grounded in the mirror self-recognition phase, in which the world is primarily understood through embodied experience. Thinking at this stage relies more on intuitive processing than on abstract conceptualization. Language acquisition is therefore dominated by vocabulary referring to concrete objects.

#### 4.3.2 Language awareness and acquisition during the stage of affective dialogue

According to Piaget’s theory of cognitive development, cognitive structures and language processing in the preconceptual substage rely heavily on sensorimotor experience, with limited capacity for symbolic representation. This helps explain why, as shown in the earlier observation, Dundun could fluently sing “Nestling into mama’s embrace, I’m happy forever” without understanding its semantic content. At that time, she appeared to process the phonological sequence and prosodic features of the lyric rather than its conceptual meaning. However, at 27 months, she patted her chest and said, “Mama is here,” indicating the initial emergence of an embodied understanding of the metaphorically complex compound “embrace.”

In Mandarin Chinese, the word “embrace” consists of two morphemes, “huái” (怀) and “bào” (抱). The original meaning of “huái” (怀) refers to the area in front of the chest, and is extended to denote the “inner mind,” thereby functioning as a metaphorical term with emotional connotations. The semantic structure of “embrace” can be characterized as follows: (i) it exhibits both verbal and nominal functions; (ii) it encodes a multi-layered metaphorical mapping from concrete bodily experience to abstract meanings; (iii) it is predominantly associated with positive affect; and (iv) as a spatialized concept, it supports multiple metaphorical extensions. Faced with this semantic complexity, Dundun’s understanding progressed from lack of attention to non-comprehension and ultimately to embodied interpretation. In Case 3, when asked by her grandmother, “What is mama’s embrace?,” Dundun replied, “Mama’s embrace is mama.” This response indicates that her understanding had moved beyond a purely spatial or referential interpretation of the term and entered a deeper level of conceptual processing. By integrating affective semantic features such as warmth, care, and security, she effectively mapped “mama’s embrace” onto “mama” herself, demonstrating an emerging ability to reorganize abstract meaning based on embodied and emotional experience. This pattern provides evidence of cognitive development during the stage of affective dialogue. At this stage, her understanding of “embrace” was grounded in accumulated embodied experiences, specifically repeated experiences of being held and associated feelings of warmth, security, and dependence. As a result, she interpreted “embrace” as an experience located within her own bodily space, reflecting a transitional form of understanding that integrates concrete and abstract elements. This progression highlights the role of embodied experience in mediating the acquisition of abstract concepts in early language development.

During the stage of affective monologue, children primarily acquire concrete lexical items and establish word-object correspondences alongside self-referential language awareness. With the transition to affective dialogue, corresponding to the early formation of egocentric awareness, the development of symbolic function and recursive language abilities enables children to incorporate abstract concepts into their linguistic system. Through egocentric speech, children begin to develop metalinguistic awareness and self-regulatory capacities, reflecting the coordinated development of cognition, language, and social interaction.

### 4.4 Multidimensional projection of egocentric consciousness and the expansion of cognitive categories driven by bidirectional interaction

During the stage of bidirectional interaction, characterized by an emerging expectation of others’ feedback, Dundun adapted the song lyrics by replacing “policeman” with “grandmother” (Case 4). In broader social contexts, “policeman” represents social norms and institutional authority. This substitution reflects a symbolic remapping, whereby an institutional symbol is recoded as an affective one. At the cognitive level, this transformation indicates that Dundun assigns her grandmother the role of an authority figure within a socio-ethical framework.

In Case 5, when Dundun received feedback that conflicted with her expectation (i.e., that the ice cream had melted), this elicited frustration. In response, she connected her sensory experiences (the heat of the sun and the melting of the ice cream) with her semantic understanding of “bossy,” thereby constructing a coherent causal chain. At the same time, this utterance reflects a cognitive shift in which the physical sun is transformed into an anthropomorphic figure (“Grandpa Sun”) endowed with human-like attributes and affective significance. This suggests that, under the conditions of bidirectional interaction, she engaged in a sequence of processes, including self-perception, projection onto external objects, internal emotional response, linguistic encoding, and meaning construction. Her egocentric awareness thus expanded outward—from the self to an emotional object and further to a physical entity—forming a multidimensional projection of self-centered cognition. The shift in attentional focus, from “I cannot eat the ice cream” to “Grandpa Sun is bossy,” marks a transition from self-oriented representation to socially mediated symbolic expression. Such a transition can be interpreted as a developmental shift from a “perceptual self” to a “symbolic self” (Mead, 1934), which illustrates how self-centered awareness and language production reinforce one another and further signals the child’s progression toward the stage of inner speech. In other words, social feedback embedded in bidirectional interaction provides opportunities for cognitive extension, enabling a shift from immediate self-expression (e.g., the desire to eat ice cream) to causal reasoning (i.e., asking “why”), and further to the imaginative construction of a broader experiential world (e.g., the sun and the moon). This pattern suggests that the affective demands of bidirectional interaction enable the child to generate cross-domain embodied experiences while simultaneously expanding her cognitive horizon.

## 5 Conclusion

Building on foundational work in developmental psychology, this study, based on a case analysis of a two-year-old child, suggests that children’s emotional expression undergoes a transition from affective monologue to affective dialogue as they develop from self-recognition to egocentric awareness. The stage of affective monologue corresponds to the phase of self-recognition and aligns with Amsterdam’s mirror self-recognition stage, whereas affective dialogue corresponds to the early emergence of egocentric awareness described in Piaget’s theory of cognitive development.

Emotional expression in children, as observed in this study, constitutes constitutes a key behavioral characteristic associated with the stages of self-recognition and egocentric awareness. It can be categorized into two forms: affective monologue and affective dialogue. Affective monologue characterizes the self-recognition stage, whereas affective dialogue marks the initial formation of egocentric awareness. At this stage, the systematic functioning of children’s emotional expression begins to emerge. Affective dialogue stimulates multidimensional projections of egocentric awareness, generates multiple layers of cognitive linkages, expands cognitive categories, and facilitates the development of social cognition.

The theoretical framework proposed in this study offers a micro-level account of how children’s emotional expression develops across stages, illuminating the processes of emotional socialization and the internalization of egocentric speech. Positioned between basic emotional expression and more advanced forms of emotional expression, it offers an initial exploration of the “meso-level” of emotional development. The limitations of single-case research are acknowledged; therefore, future studies should draw on larger samples and multi-dimensional data sources to further validate the stage-specific characteristics and functional significance of children’s emotional expression. Such work may contribute to the construction of an integrated “emotion-language co-development model,” with potential implications for early intervention and educational practice.

## Data Availability

The original contributions presented in the study are included in the article/Supplementary material, further inquiries can be directed to the corresponding author.
