# Supplementary material for: Patterns of emotional expression during the formation of egocentric awareness in early childhood: a case study
Source: Front Psychol. 2026 Apr 20;17:1756750. doi: 10.3389/fpsyg.2026.1756750 (PMC13154853; doi:10.3389/fpsyg.2026.1756750)
Supplement: Supplementary file 1 [file Supplementary_file_1.pdf]

Supplementary Material 1

Case 1: Adaptation of Nursery Rhyme Themes (18 - 23 months)

18 months: Dundun was able to hum the nursery rhyme “A Mama’s Love is the Best in the World” fluently.

19 months: She replaced the word “mama” with “papa” in the opening line.

22 months: She replaced all three instances of “mama” with “papa”, singing:

“A papa’s love is the best in the world; With papa, I’m a treasure; Nestling into papa’s embrace, I’m happy forever” (in the absence of her father).

23 months: She produced a new version replacing “mama” with “gege” (older brother), again in his absence.

Case 2: Daily Conversation with the Maternal Grandmother at 26 Months (Understanding of Reference)

Grandmother: “Who is the ‘mama’ in ‘A Mama’s Love is the Best in the World’?”

Dundun: “My mama.”

G: “And who is ‘papa’ in ‘A papa’s love is the best in the world’?”

D: “My papa.”

G: “Who is the ‘treasure’?”

D: “Me. I am mama’s treasure.”

G: “Who is ‘happy’ when singing ‘happy forever’?”

No response; the child did not yet demonstrate an understanding of the abstract concept of “happy.”

Case 3: Concrete Interpretation of an Abstract Concept (27 months)

During a nursery rhyme activity with her maternal grandmother, Dundun sang the line “Nestling into mama’s embrace, I’m happy forever.” She then patted her chest and said, “Mama is here.” When asked, “What is mama’s embrace?”, Dundun replied, “Mama’s embrace is mama.”

Case 4: Song Adaptation — “One Cent” (28 months)

Dundun entered the kitchen, handed a coin to her grandmother, looked directly at her, and sang: “I picked up one cent on the roadside and handed it to grandma.” instead of the original “to the policeman”. Grandmother responded, “Dundun did a good job. You’re a good girl.” Dundun joyfully threw herself into her grandmother’s arms.

Case 5: The “Ice Cream” Event (28 months)

During an outing, Dundun wanted another ice cream. Her father said it had melted. She protested: “Grandpa Sun is too bossy! Making the day so hot! The ice cream melted! Grandpa Sun is too bossy! Let the moon come out!”
